# An ensemble-based Cox proportional hazards regression framework for predicting survival in metastatic castration-resistant prostate cancer (mCRPC) patients

**DOI:** 10.12688/f1000research.8226.1

**Published:** 2016-11-16

**Authors:** Richard Meier, Stefan Graw, Joseph Usset, Rama Raghavan, Junqiang Dai, Prabhakar Chalise, Shellie Ellis, Brooke Fridley, Devin Koestler

**Affiliations:** 1 Department of Biostatistic, University of Kansas Medical Center, Kansas City, KS, USA; 2 Department of Health Policy and Management, University of Kansas Medical Center, Kansas City, KS, USA

**Keywords:** Ensemble-based modeling, prostate cancer, DREAM challenge, mCRPC, survival analysis

## Abstract

From March through August 2015, nearly 60 teams from around the world participated in the Prostate Cancer Dream Challenge (PCDC). Participating teams were faced with the task of developing prediction models for patient survival and treatment discontinuation using baseline clinical variables collected on metastatic castrate-resistant prostate cancer (mCRPC) patients in the comparator arm of four phase III clinical trials. In total, over 2,000 mCRPC patients treated with first-line docetaxel comprised the training and testing data sets used in this challenge. In this paper we describe: (a) the sub-challenges comprising the PCDC, (b) the statistical metrics used to benchmark prediction performance, (c) our analytical approach, and finally (d) our team’s overall performance in this challenge. Specifically, we discuss our curated, ad-hoc, feature selection (CAFS) strategy for identifying clinically important risk-predictors, the ensemble-based Cox proportional hazards regression framework used in our final submission, and the adaptation of our modeling framework based on the results from the intermittent leaderboard rounds. Strong predictors of patient survival were successfully identified utilizing our model building approach. Several of the identified predictors were new features created by our team via strategically merging collections of weak predictors. In each of the three intermittent leaderboard rounds, our prediction models scored among the top four models across all participating teams and our final submission ranked 9 ^th^ place overall with an integrated area under the curve (iAUC) of 0.7711 computed in an independent test set. While the prediction performance of teams placing between 2 ^nd^- 10 ^th^ (iAUC: 0.7710-0.7789) was better than the current gold-standard prediction model for prostate cancer survival, the top-performing team, FIMM-UTU significantly outperformed all other contestants with an iAUC of 0.7915.  In summary, our ensemble-based Cox regression framework with CAFS resulted in strong overall performance for predicting prostate cancer survival and represents a promising approach for future prediction problems.

## Introduction

Today, prostate cancer is one of the most prevalent cancers afflicting men in the Western world. In addition to the prevalence of this disease, the mortality rates for prostate cancer ranked fifth among the most common causes of cancer death worldwide in 2012 ( http://www.cancerresearchuk.org/). In the US alone, approximately 137.9 out of 100,000 men were diagnosed with prostate cancer each year from 2008–2012, with an average annual mortality rate of 21.4 out of 100,000 men. ( http://www.seer.cancer.gov/statfacts/html/prost.html). According to the Cancer Prevalence and Cost of Care Projections, the total annual cost of prostate cancer in 2016 has been estimated at 14.3 billion dollars ( http://www.costprojections.cancer.gov/).

Over the course of the last decade in the US, approximately 15% of prostate cancer cases were initially diagnosed with metastatic disease (stage IV). Androgen deprivation therapy (ADT) is the established treatment for these cases, but one third of patients develop resistance and their disease progresses to metastatic castrate-resistant prostate cancer (mCRPC) ( https://www.synapse.org/ProstateCancerChallenge). Treatment of mCRPC has been historically challenging, and while docetaxel – the current front-line therapy for mCRPC – has been effective at improving mCRPC survival at the population level, a significant fraction of patients do not respond to treatment or prematurely discontinue treatment due to adverse events (AE) ^1^, leading to substantial variation in the individual outcomes between mCRPC patients. For this reason, and because of the tremendous personal, societal, and economic burden associated with this disease, there is significant interest both in the identification of individual predictors for mCRPC prognosis as well as the development of prognostic models that can be used to identify high-risk mCRPC patients.

In a recent publication ^2^, Halabi *et al.*, utilized data from a phase III trial consisting of over one thousand mCRPC patients to develop and test a prognostic model for overall survival among patients receiving first-line chemotherapy. The time dependent area under the curve (tAUC) was > 0.73 in both testing and independent validation data sets, suggesting strong performance of the Halabi *et al.* model for identifying low- and high-risk mCRPC patients. Notwithstanding the significant advances made by Halabi *et al.*, and others toward the development of accurate prognostic models for mCRPC outcomes ^2– 4^, there remains ample room for improved prediction performance.

Motivated by the potential to further improve existing risk-prediction tools along with growing worldwide burden of prostate cancer, the Prostate Cancer Dream Challenge was launched in March 2015 and included the participation of nearly 60 teams from around the world. The Prostate Cancer Dream Challenge was composed of two distinct sub challenges; in sub challenge 1, teams competed in the development of prognostic models for predicting overall survival based on baseline clinical variables, whereas the objective of sub challenge 2 involved the development of models to predict short-term treatment discontinuation of docetaxel (< 3 months) due to adverse events (AE). To assist in the development and testing of prediction models, approximately 150 variables collected on over 2,000 mCRPC patients treated with first-line docetaxel in one of four different phase III clinical trials were used. Three of the four trials were combined to generate the training data set, which was used for model-building and development, while data from the remaining trial were withheld from challenge participants and used as an independent test set to evaluate each of the submitted models ^5^.

In the present manuscript, we focus exclusively on our methodological approach to sub challenge 1. Broadly speaking, the first step of our team’s approach to sub challenge 1 involved an initial screening of the data: data cleaning and processing, creation of new variables from existing data, imputation and/or exclusion of variables with missing values, and normalization to standardize the data across trials. The final “cleaned and standardized” training data was then used to fit to an ensemble of multiple Cox proportional hazards regression models whose constituent models were developed using curated, ad-hoc, feature selection (CAFS). Models developed by our team were subjected to internal cross-validation within the training data set to identify instances of model overfitting and to assist in further refinements to our prediction models. The source code utilized for our approach can be accessed via the Team Jayhawks Prostate Cancer Dream Challenge project web page ( https://www.synapse.org/#!Synapse:syn4214500/wiki/231706) or directly via the GitHub repository webpage ( https://github.com/richard-meier/JayhawksProstateDream).

## Materials and methods

### Data

A detailed description of the datasets used in this challenge can be found on the Prostate Cancer Dream Challenge web page ( https://www.synpase.org/ProstateCancerChallenge). Briefly, the training set originated from the ASCENT-2 (Novacea, provided by Memorial Sloan Kettering Cancer Center), MAINSAIL (Celgene) and VENICE (Sanofi) trials ^6– 8^. For the 1600 patients in the training data, baseline covariate information and clinical outcomes (i.e. time to death and time to treatment discontinuation) were provided to participating teams for the purposes of model development and training. Although baseline covariate information for a subset of patients in the ENTHUSE-33 (AstraZeneca) trial ^9^ scoring set was provided to participating teams (n = 157), the clinical outcomes for each of these patients were censored and withheld from teams throughout the duration of the challenge. Specifically, the ENTHUSE-33 data set (n = 470) was split into two disjoint sets that consisted of 157 and 313 patients. Whereas an undisclosed randomly selected subset of the 157 patients was used for model evaluation in each intermittent leaderboard round, the remaining 313 patients were withheld completely from participating teams and used only in the final scoring round.

### Preprocessing

All aspects of our approach, from data preprocessing to model development and cross-validation, were implemented using R version 3.2.1 (2015-06-18) ( https://www.r-project.org/). Baseline covariate information on subjects comprising the training data were reformatted and normalized according to the type of variable (i.e., categorical, ordinal, numeric) and feature type (i.e., medical history, laboratory values, etc). Cleaned and normalized baseline features were then used to derive additional novel risk predictors. ( https://github.com/richard-meier/JayhawksProstateDream/blob/master/dataCleaningMain.R)

Several groups of binary variables representing patient specific clinical information and prior medical history reported on patients were merged into new features. Three different merging types were explored: “logical or”, regular summation, and z-score weighted summation. For the latter, each individual feature in the training set was fit against survival time with a Cox proportional hazards model and their resulting z-scores were used to derive weights that were proportional to each variable’s strength of association with survival ( https://github.com/richard-meier/JayhawksProstateDream/blob/master/deriveHardcodedWeights.R). Summation variables were created for 3 main categories: medical history information, prior medication information and metastasis information. For each of these categories, new variables generated by merging specific subcategories (i.e. protective, harmful, total, visceral, etc.) were created.

A participant’s target lesion volume (TLV) was generated by multiplying each target lesion by its size, followed by summing over all lesions within that participant ( https://github.com/richard-meier/JayhawksProstateDream/blob/master/src/lesion_volume.R). To impute the TLV for the ASCENT-2 trial, we calculated the average TLV per lesion within individuals who survived or died in the MAINSAIL or VENICE trials, and multiplied these separate averages by the number of non-bone lesions found in the ASCENT-2 data. To classify whether for each category a feature was in the subcategory “protective” or “harmful”, their z-scores, when individually fitting against the outcome, were used. A feature was labeled "protective" if its z-score was greater than 1.64 and "harmful" if its z-score was smaller than -1.64.

Principal component analysis (PCA) was used to split numerical laboratory values into components that best explained their variation (see above: “deriveHardcodedWeights.R”). The top PCs were then treated as new features. In order to address issues or findings involving some specific variables, additional features were created: The ECOG performance status score was both included as continuous and categorical variable. Age groups were also recoded as an ordinal age risk variable for which 0 represented patients older than 75 years, 1 represented patients younger than 65 years and 2 represented patients with ages between 65–75 years. The latter was motivated by our observation of a non-linear trend between age and survival time.

Race was recoded into a binary variable where 1 referred to patients labeled as “white” or “other” and 0 represented patients that did not fall into one of those two categories (e.g. "black", "asian", etc.). The features “harm_pro” and “harm_pro2” were created by fitting the summation variables of the medical history subgroups “harmful” and “protective” against the outcome and obtaining the z-scores of these subgroup summation variables. The difference between the two features was that harm_pro exclusively fitted the two summation variables, whereas harm_pro2 also utilized a set of important predictor variables for the initial fit. The two z-score weighted sums (corresponding to the two sets of features utilized for the previously mentioned fit) of these summation variables then correspond to the two new features. ( https://github.com/richard-meier/JayhawksProstateDream/blob/master/src/add_additional_features.R)

### Model building and feature selection

**Figure 1.**
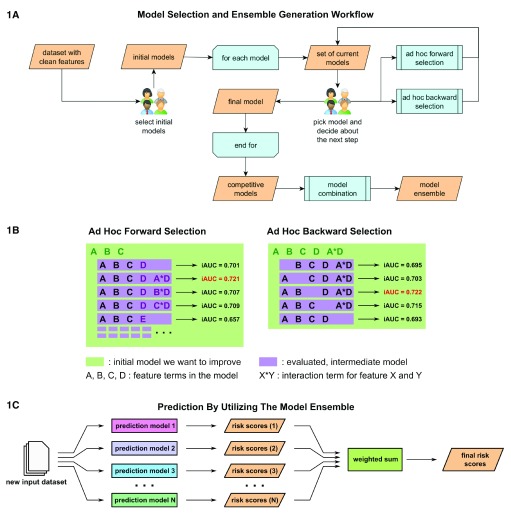
Model building and model ensemble utilization. ( **1A**) Competitive prediction models were built individually by a curated, ad-hoc feature selection procedure. In each step researchers picked a new best model from the set of current models based on an optimization criterion and decided how it would be processed. ( **1B**) Models were optimized by either forward selection, in which a new feature was added, or backward selection, in which a feature that had become obsolete was removed. Both selection methods generated a set of new models for which performance was predicted via in-depth cross-validation. ( **1C**) Once a variety of competitive prediction models had been created, models were combined into an ensemble, which averaged their individual predictions in order to increase performance.

Our methodological framework utilized an ensemble of Cox proportional hazards regression models that were found to be individually competitive in predicting survival. For each patient, the ensemble-based risk scores were generated as a weighted sum of the individually estimated risk scores from separate Cox-regression models, fit using the “coxph” function in the “survival” R-package ^10^ ( Figure 1C). Feature selection among the competitive risk-prediction models that constituted our ensemble was undertaken by a method we call curated, ad-hoc, feature selection (CAFS). This method attempts to maximize the prediction performance of a given model by iteratively including and excluding features from a baseline initial model. The method is greedy in the sense that in each step of the algorithm, only the model candidates that achieve the current "local best" performance are selected. Each iteration started with a group of experts making two executive decisions based on a set of possible model candidates for which performance was evaluated in prior iterations. First, one model was nominated as the best current model and a decision was made whether to expand or shrink the model, or terminate the procedure and keep the model, in its current form ( Figure 1A). Choosing the current best model was guided by a candidate’s estimated performance, performance of the previous best model, as well as knowledge of the researchers as to whether the form and components of a given model were reasonable in the context of the problem at hand. An example for the latter case would be that a newly introduced interaction term between completely unrelated features might be rejected after evaluation, even though it technically achieved the current best performance.

Model reduction was done via ad-hoc backward selection ( Figure 1B). In this procedure a set of new models was generated by individually excluding each parameter or feature present in the current model. For each of these models, performance was evaluated based on a previously chosen optimization criterion, i.e., integrated time-dependent area under the curve (iAUC). The criterion was estimated via a cross-validation procedure in which the training set was repeatedly split into two random subsets of a fixed size. The first subset was used to estimate parameters of a given model, whereas the second subset was used to predict the outcome using the previously estimated parameters and to calculate the optimization criterion based on comparing the prediction with the true outcome. In our study, we utilized two-thirds for the parameter estimation subset, i.e., first subset, while the remaining one third comprised the second subset. The average of the calculated optimization criterion values, obtained from all random splits, served then as a performance estimate. We used 10,000 cross-validation steps for each model in our study to ensure stability of the average performance. The new models and performance estimates were then used as the basis for subsequent iterations.

Expansion of a model was accomplished using an ad-hoc forward selection procedure ( Figure 1B). In this procedure several new models were created for each feature within the feature space. Each subset of new models contained one base model that included only main effect terms for new features, i.e., no interaction terms included. All other models in the subset further expanded this base model by individually introducing an interaction term with each element already in the previous best model.

Performance of each new model was again assessed via the cross-validation procedure. Since this step iterated over the feature space, it created a large amount of different models. To make this step computationally feasible, the number of cross-validation iterations had to be reduced. In our study, 500 cross-validation steps per new model were utilized. ( https://github.com/richard-meier/JayhawksProstateDream/blob/master/src/modelTuning.R)

Finally, since the variances of these performance estimates were much higher than in the shrinkage step, the top 30 performing models were chosen and performance was re-estimated via 10,000 fold cross-validation. This set of new models and performance estimates was then used in the next iteration. Once iterations provided only marginal performance increases, the procedure was terminated and a final model was declared. Different models for the ensemble were found either by choosing different intermediate models as the current best and branching off a certain path, or by choosing different initial models.

### Model evaluation

Each of the sub challenges in the Prostate Cancer Dream Challenge had its own prediction scoring metrics. In sub challenge 1A, participants were asked to submit a global risk score and time dependent risk scores, optimized for 12, 18 and 24 months. These risk scores were evaluated utilizing two scoring metrics: a concordance index (cIndex), and an integrated time dependent area under the curve (iAUC; 6–30 months). The time specific risk scores were assessed using AUC’s computed using Hung and Chiang’s estimator of cumulative AUC ^11^. In sub challenge 1B, participants were asked to predict the time to event (death). The predictions of time to event were scored utilizing the root mean squared error (RMSE), using patients with known days to death.

When applying CAFS, we utilized the iAUC calculated from the predicted risk scores as an optimization criterion. This measure was also used by the challenge organizers for performance assessment in the scoring rounds for sub challenge 1A. While participants were asked to predict the risk score for overall survival based on patients' clinical variables, they were also tasked to predict the time to event (TTE) in sub challenge 1B. We used the risk score for each patient to model the TTE:

*TTE _i_* = *f*( *riskScore _i_*) + *∈ _i_*

Where *riskScore _i_* corresponds to the risk score calculated in sub challenge 1A for the i ^th^ patient and *f* is an unknown smoothing function. We estimated *f* using a Generalized Additive Model (GAM) via the “gam” function within the “mgcv” package in R ^12^. When regressing TTEs on risk we used only the subset of individuals who died.

## Results

The principal component analysis with all laboratory values revealed that the first principal component was highly correlated with patient survival. Furthermore, across all laboratory values, only a subset of six features (baseline levels of: albumin, alkaline phosphatase, aspartate aminotransferase, hemoglobin, lactate dehydrogenase and prostate specific antigen) contributed significantly to explaining the variation in said first component. Thus, in the first PC only these six laboratory values were used during model building and development. In addition to the first principal component, several other newly created metavariables were identified as clinically relevant predictors by our model building procedure. Three z-score weighted sums merging metastases locations, medical history and prior medication were included in our prediction models. The “logical or” merged variable, whether or not a patient had any known medical history issues, was also utilized. The protective versus harmful subcategorization was only included in the models in the form of the sum of protective medical history features. However, this category only included a single feature, vascular disorders (yes/no).

**Figure 2.**
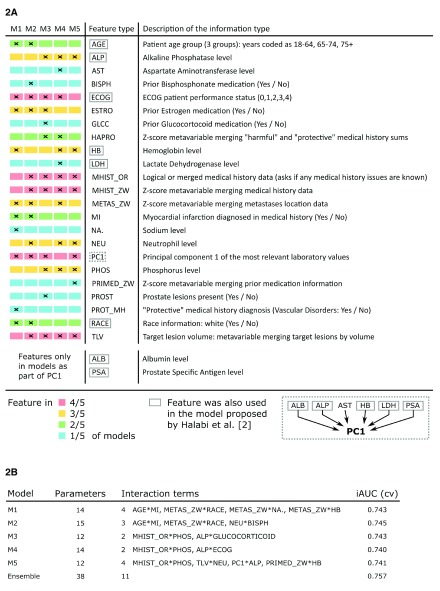
Generated models utilized in the final challenge submission. ( **2A**) The ensemble consisted of five different models, M1 to M5, which ended up sharing many feature types even though they were individually generated under different conditions. ( **2B**) All models made use of a similar number of parameters and achieved comparable performance in cross-validation. Performance further increased when using the model ensemble.

We developed 5 competitive prediction models (M1 – M5) that were used in our Cox proportional hazards regression ensemble ( Figure 2). All models were developed by either refining a previous model via CAFS or by building a model from the bottom up via CAFS. M1 used the best model found by manually selecting promising features as its initial model. M2 used an intermediate model from the CAFS procedure of M1 to deliberately branch off and provide a similar, yet different model. M3 and M5 were both built by using an initial model solely utilizing the strong predictors target lesion volume and principal component 1, but branching off in early iterations. M4 was built by using an initial model utilizing target lesion volume and the alkaline phosphatase level under the restriction that principal component 1 was excluded from the feature space.

While no single feature was utilized in every model M1–M5, five different features were shared between four models, six features between three models, four features between two models and eight features were unique to a model ( Figure 2A). Each model had at least one unique feature. Between two and four interaction terms (two-way interaction terms) were present in all of the observed models ( Figure 2B). One interaction was shared between the models M3, M4 and M5, while two interactions were shared between two models M1 and M2. Including components of newly derived features, eight features that were included in the original model by Halabi *et al.* in some form, were also utilized in the model ensemble. In total, the ensemble contained 38 coefficients, out of which 11 were pairwise interaction terms across all models.

The estimated iAUC during performance assessment was found to be stable up to approximately three decimals when using 10,000 fold cross-validation. Similar estimated performance within the range of 0.005 iAUC difference was achieved between the competitive prediction models, the highest total iAUC being 0.745. Optimal weights were chosen based on randomly initializing weights 100 times and estimating performance. Performance tended to be optimized the smaller the maximum pairwise difference between weights in an ensemble was. The best possible performance was estimated when choosing equal weights for all models. This ensemble was chosen as the best model. Utilizing the ensemble led to an estimated performance increase of 0.012 iAUC.

**Figure 3.**
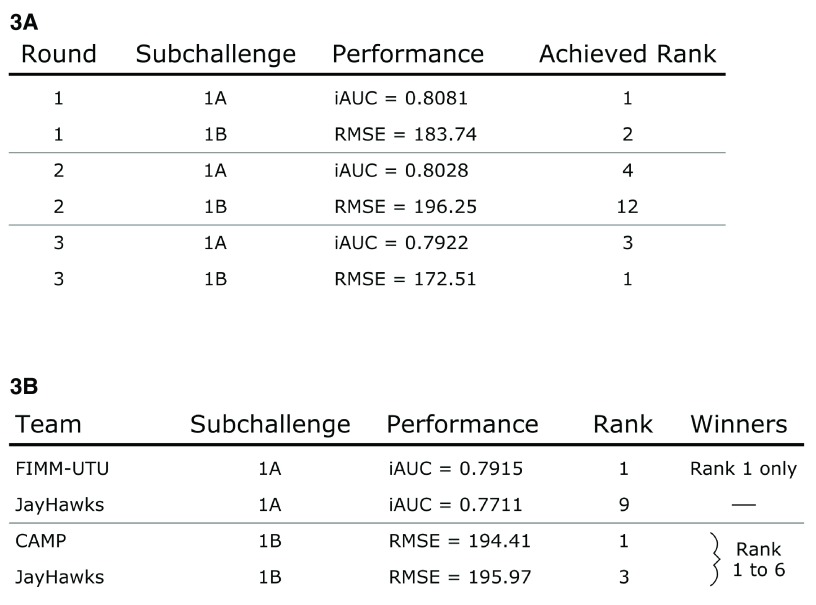
Team performance during the challenge. ( **3A**) Submitted models were consistently ranked at the top of the leaderboards during the scoring rounds before the final submission. Models build via the CAFS procedure were submitted starting with the second leaderboard round. ( **3B**) The final challenge submission made use of the described model ensemble approach and was placed at rank 9 in sub challenge 1A and at rank 3 in sub challenge 1B.

During the three leaderboard rounds the team explored and submitted various methodologies. Top performing submissions were always Cox proportional hazards models that outperformed more sophisticated approaches such as generalized boosted regression models and random survival forests. From scoring round 2 onward, single models utilizing CAFS were also submitted. In all intermittent leaderboard rounds, at least one of our submitted entries ranked among the top 4 performing models of sub challenge 1A ( Figure 3A). In sub challenge 1B, at least one submission was within the top 3 performing models, with the exception of the second leaderboard round were our best model ranked number 12. Our models achieved performances ranging from 0.792 to 0.808 iAUC in 1A and from 172.51 to 196.25 RMSE in 1B. In the final scoring round, team FIMM-UTU ^5^ significantly outperformed all other contestants with an iAUC of 0.7915 ( Figure 3B). Our submission for 1A that utilized the CAFS ensemble achieved rank 9 with an iAUC of 0.7711. The performances of teams ranking from 2 ^nd^ to 10 ^th^ were very similar. While the difference in performance between rank 1 and 2 was 0.0126 iAUC, the difference in performance between our method and rank 2 was only 0.0078 iAUC. Our submitted model ensemble also successfully outperformed the previous best model by Halabi *et al.* ^2^, which was placed at rank 36 with an iAUC of 0.7429. Sub challenge 1B was won simultaneously by 6 teams out of which our method achieved rank 3.

## Discussion

Many feature types present in the original model by Halabi *et al.* ^2^ were also independently picked up and retained by CAFS. This solidifies the idea that these might be key components influencing survival. Considering that five out of these eight were also involved in the first principal component, which was one of the strongest predictors, does also support this. Another set of potentially interesting predictors are those shared between three or more models.

It is debatable whether the fact that a lot of overlap exists between the various sub-models points towards the validity of selected features and the developed approach, or a potential bias in the feature selection procedure. However, the former appears more likely in the light of the approach’s good performance on new data in the competition.

The included interaction terms are difficult to interpret. There is no guarantee that an interaction is modeling a direct relationship and some terms might be artifacts of higher order interactions or confounding issues. Also, when solely including terms into the model based on the optimization criterion in each step of CAFS, there is a bias to include interaction terms. Since they introduce more parameters into the model than a main effect, they have more opportunity to improve the model within each step, even though including two different main effects in a row might be more beneficial. While our team was aware of this issue and cautious with the selection of sub-models, this still leaves potential for making suboptimal choices. This weakness could potentially be addressed in the future by switching to a parameter count based iteration, rather than a feature type based iteration.

The performed recoding of the age groups is still problematic. Intuitively, it does not make sense that the order “oldest, youngest, in-between” would be related to the outcome when disease progression usually worsens with age. A possible explanation might be that the oldest patient group is confounded with a subset of people that are resistant to the disease and have already survived for a long time. Further research is required to validate this effect.

Overall the presented method successfully built a robust predictor for the target outcome. Evidence for this is provided by the fact that the estimated performance via in-depth cross validation (iAUC = 0.757) was close to the reported performance on the larger, final leaderboard set (iAUC = 0.771) and the fact that our models were among the top performing candidates throughout the entire challenge. It should also be highlighted that the required human intervention in each selection step gives the team of researchers a lot of control, which can be very useful to introduce knowledge about the feature space into the selection process. An example of this benefit is that despite the pointed out weakness in the implementation, the team was able to account for it by rejecting inclusions of interactions that did not have a great enough impact. If desirable, early branches of the selection process can be tailored towards features with a known connection to the outcome, when multiple feature inclusions provide similar performance benefits.

## Conclusion

The presented method generated a model ensemble that was able to outperform the previous best efforts to predict survival in prostate cancer patients. The developed model ensemble also successfully competed with the top performing research teams in the Prostate Cancer Dream Challenge and was among the winning teams in sub challenge 1B. We attribute this success to careful data cleaning, our efforts to derive novel features and the fact that skeptic, human decision making is integral to each iteration of the curated ad-hoc feature selection. Due to its general applicability to model building, especially in exploratory settings, the approach is promising in being useful for researchers around the world. Future studies will need to validate the presented, potentially disease associated features and potential weaknesses in the CAFS procedure should be investigated and addressed.

## Data availability

The data referenced by this article are under copyright with the following copyright statement: Copyright: © 2016 Meier R et al.

Data associated with the article are available under the terms of the Creative Commons Zero "No rights reserved" data waiver (CC0 1.0 Public domain dedication). 

The Challenge datasets can be accessed at: https://www.projectdatasphere.org/projectdatasphere/html/pcdc

Challenge documentation, including the detailed description of the Challenge design, overall results, scoring scripts, and the clinical trials data dictionary can be found at: https://www.synapse.org/ProstateCancerChallenge

The code and documentation underlying the method presented in this paper can be found at: http://dx.doi.org/10.5281/zenodo.49063 ^13^

## References

[1] Schallier D Decoster L Braeckman J: Docetaxel in the treatment of metastatic castration-resistant prostate cancer (mCRPC): an observational study in a single institution. *Anticancer Res.* 2012;32(2):633–41. 22287756

[2] Halabi S Lin CY Kelly WK: Updated prognostic model for predicting overall survival in first-line chemotherapy for patients with metastatic castration-resistant prostate cancer. *J Clin Oncol.* 2014;32(7):671–7. 10.1200/JCO.2013.52.3696 24449231 PMC3927736

[3] Chang K Kong YY Dai B: Combination of circulating tumor cell enumeration and tumor marker detection in predicting prognosis and treatment effect in metastatic castration-resistant prostate cancer. *Oncotarget.* 2015;6(39):41825–41836. 10.18632/oncotarget.6167 26497689 PMC4747191

[4] van Soest RJ Templeton AJ Vera-Badillo FE: Neutrophil-to-lymphocyte ratio as a prognostic biomarker for men with metastatic castration-resistant prostate cancer receiving first-line chemotherapy: data from two randomized phase III trials. *Ann Oncol.* 2015;26(4):743–9. 10.1093/annonc/mdu569 25515657

[5] Guinney J Wang T Laajala TD: Prediction of overall survival for patients with metastatic castration-resistant prostate cancer: development of a prognostic model through a crowdsourced challenge with open clinical trial data. *Lancet Oncol.* 2016 10.1016/S1470-2045(16)30560-5 PMC5217180 27864015

[6] Scher HI Jia X Chi K: Randomized, open-label phase III trial of docetaxel plus high-dose calcitriol versus docetaxel plus prednisone for patients with castration-resistant prostate cancer. *J Clin Oncol.* 2011;29(16):2191–2198. 10.1200/JCO.2010.32.8815 21483004

[7] Petrylak DP Vogelzang NJ Budnik N: Docetaxel and prednisone with or without lenalidomide in chemotherapy-naive patients with metastatic castration-resistant prostate cancer (MAINSAIL): a randomised, double-blind, placebo-controlled phase 3 trial. *Lancet Oncol.* 2015;16(4):417–425. 10.1016/S1470-2045(15)70025-2 25743937

[8] Tannock IF Fizazi K Ivanov S: Aflibercept versus placebo in combination with docetaxel and prednisone for treatment of men with metastatic castration-resistant prostate cancer (VENICE): a phase 3, double-blind randomised trial. *Lancet Oncol.* 2013;14(8):760–768. 10.1016/S1470-2045(13)70184-0 23742877

[9] Fizazi K Higano CS Nelson JB: Phase III, randomized, placebo-controlled study of docetaxel in combination with zibotentan in patients with metastatic castration-resistant prostate cancer. *J Clin Oncol.* 2013;31(14):1740–1747. 10.1200/JCO.2012.46.4149 23569308

[10] Therneau TM: A Package for Survival Analysis in S.version 2.38,2015 Reference Source

[11] Hung H Chiang CT: Estimation methods for time-dependent AUC models with survival data. *Can J Stat.* 2010;38(1):8–26. 10.1002/cjs.10046

[12] Wood SN: Fast stable restricted maximum likelihood and marginal likelihood estimation of semiparametric generalized linear models. *J R Stat Soc Series B Stat Methodol.* 2011;73(1):3–36. 10.1111/j.1467-9868.2010.00749.x

[13] Chalise P Dai J Ellis S: JayhawksProstateDream: First release (PCDC submission). *Zenodo.* 2016 Data Source

