# Supplementary material for: An ensemble-based Cox proportional hazards regression framework for predicting survival in metastatic castration-resistant prostate cancer (mCRPC) patients
Source: F1000Res. 2016 Nov 16;5:2677. [Version 1] doi: 10.12688/f1000research.8226.1 (PMC5365222; doi:10.12688/f1000research.8226.1)
Supplement: Peer review history

# Peer review: Referee response for version 1

**DOI:** 10.5256/f1000research.8848.r17707

**Published:** 2017-03-20

**Authors:** Russell Greiner, Luke Kumar

**Affiliations:** 1 Department of Computing Science, University of Alberta, Edmonton, AB, Canada

The authors describe  the model they submitted to the recent “Prostate Cancer DREAM Challenge - Sub-Challenge - 1”, which was ranked among the top models in the challenge. In particular, they describe their feature selection process, which they mostly credit for their success in the challenge.  The manuscript summarized their approach and the results from the challenge in an adequate manner.  However, we have some concerns:

 Main Critique:

 Their CAFS feature selection (FS) process seems to related to the wrapper feature selection methods, but includes a human expert in the loop.  This greatly reduces the reproducibility of this work. Also, the authors have not clearly listed the guideline followed by the experts when deciding on features, which appears to further reduce the usefulness of this approach in general.

 Minor Points:

1. The final selected model from the FS process is not explicit from Figure 1B. It would also be better if the authors explicate CAFS’s boundary between expert intervention vs data-driven selection.
2. The basic algorithm embodied several assumptions -- eg, p6 mentions 6 features of PC1. Why 6?  Also why just use sum of "protective medical history features"? Why not include harmful features? We think we understand these decisions but the paper would be improved if it better motivated these various decisions.
3. Their approach of combining the ensemble with randomly selected weights seem to introduce instability to the final prediction in different runs. It would be worthwhile to give more details on this step, describing how the proposed method compares with a simple mean (or sum) and listing the motivations for this choice -- and relate this to the claim that equal weights for all models gives the best performance
4. It was great that the authors listed the results from the winning models to give the reader a good idea about the challenge itself.
5. Figure 2A was a well thought-out table, which gives the reader insights in understanding the selected features.
6. The flow diagram in Figure 1A was difficult to process as it does not show a single flow (eg: left-right or top-down).  Perhaps it would be improved with a more streamlined flow diagram of the FS process.
7. The authors mention they experimented with other survival prediction models, such as random survival forests and generalized boosted regression models.  It would be useful to show results from those models, for a better comparison.
8. The authors have used links to certain web pages in the text.  It would be more in line with academic publications if proper citations were used.

We have read this submission. We believe that we have an appropriate level of expertise to confirm that it is of an acceptable scientific standard, however we have significant reservations, as outlined above.

# Peer review: Referee response for version 1

**DOI:** 10.5256/f1000research.8848.r20536

**Published:** 2017-02-27

**Authors:** Ka Yee Yeung, Daniel Kristiyanto

**Affiliations:** 1 University of Washington, Tacoma, WA, USA

The authors documented the strategies they developed and used to predict the risk scores (Sub-challenge 1a) and survival times (Sub-challenge 1b) of prostate cancer patients as part of their participation in the DREAM 9.5 Prostate Cancer Challenge. The paper was well-written with ample of details for each step of the pipeline, with very nice figures and tables.  The models performed well and ranked well in the challenge.  However, there are still some areas that need further elaborations:

  

-  The authors mentioned missing data in the “Introduction” section.  However, the data imputation techniques used to replace the missing data, such as the lesion volume in the ASCENT-2 trial, are not described in “Materials and Methods”.  The reviewers would like to request the authors to explain what have been done to replace the missing data in all the clinical trial studies with missing data and the rationale of their strategies.
- The authors developed a “curated, ad-hoc, feature selection” (CAFS) strategy to identify predictors. The reviewers would like to request additional details on how this method selects the features, and especially on how the weights of different features are computed.
- Figure 2 showed the representation of each model and the variables involved. If the 6 considered highly correlated variables with patients survival (ALB, ALP, etc.) are already included in the first principal component (PC1) (which is used in 4/5 of the cross validation models), wouldn't it be redundant to have the same features included in the model? What were the considerations taken for this decision?
- Also related to the previous questions, model M4 was built with restrictions to not include PC1 which is claimed as the significant predictor. However, as seen in Fig 2B, this model still performed reasonably well --any observations or comments on this?

We have read this submission. We believe that we have an appropriate level of expertise to confirm that it is of an acceptable scientific standard.

# Peer review: Referee response for version 1

**DOI:** 10.5256/f1000research.8848.r17706

**Published:** 2016-12-06

**Authors:** Stephen R. Piccolo

**Affiliations:** 1 Department of Biology, Brigham Young University, Provo, UT, USA

The authors describe their participation in Subchallenge 1 of the Prostate Cancer DREAM Challenge. Their model performed well, even though it was not considered a top performer. They were creative in the way they designed their approach and tried many different options, which helped to provide insights into this particular problem as well as general strategies for model selection and optimization. Overall I was pleased with the quality of the writing and the level of detail used in the descriptions of methods and results. In particular, I like that they mentioned specific software versions and provided direct links to the code that they used for specific tasks. I did have a few questions and noticed a few gaps, which I have outlined below.

 Major points:

- The manuscript provides context about the challenge as a whole. This was helpful. For example, the authors described how their approach performed in comparison to the other approaches. However, it would have been much more insightful if the authors had provided at least a brief description of the approach used by FIMM-UTU and how that approach compared to their own and what this team might have done to perform better. In hindsight, what can they learn from this?
- The manuscript describes the CAFS approach in fairly vague terms. It makes sense that the authors used intuition to optimize the feature selection. Figure 2 also provides some insight into feature and model selection. However, it is difficult to understand much about the thought process that went into these decisions. If someone else wanted to repeat this approach, how would they go about it? Are there any general guidelines that they used in making these decisions? Maybe they could provide an example that illustrates this process. Because of this, I am hesitant to accept the claim that "the approach is promising in being useful for researchers around the world."
- The manuscript mentions imputation and dealing with missing values in a couple places. But very little, if anything, is stated in the methods (or results) about how missing values were actually handled. The authors should be more explicit in describing this.

Minor points:

- In some cases, features may have been correlated strongly with each other. For example, the z-score weighted sum values and “logical or” merged variables were derived from the same underlying data. Did the authors account for these dependencies in their models in any way? If so, how? 
- The authors used the class labels extensively in the training set to optimize their models. For example, their z-scores were generated based on the class labels, and they trained a large number of different models on the same data set. Thus it is impressive that their iAUC values generalized as well as they did on the validation set. However, it was unclear (or perhaps I missed it) whether the authors set aside any part of the training set as a pseudo-validation set. Figure 1C suggests that they did, but I didn't see any explicit explanation of this.
- For the " weighted sum" approach, it was a bit unclear exactly how the weights were calculated. In addition, the manuscript states that, "Optimal weights were chosen based on randomly initializing weights 100 times and estimating performance." What range of weights were used and how were they varied?
- The authors state that, "Different models for the ensemble were found either by choosing different intermediate models as the current best and branching off a certain path, or by choosing different initial models." At an abstract level, this makes sense, but it is hard to know exactly what this means. It would help to be more explicit on this part.
- In the Discussion, it says, "Another set of potentially interesting predictors are those shared between three or more models." But it is unclear what these predictors are (or perhaps I missed it). Mentioning these predictors explicitly would  be helpful.
- It's a little confusing to have source code in two different locations (Zenodo and GitHub). I'd suggest just pointing people to Zenodo since the data files are there, in addition to the code. Or maybe the two are integrated? But again, if that is the case, I would suggest just using one or the other.
- I am not sure you really need to mention the top-performing team in the abstract. My recommendation would be to focus the abstract more on your solution rather than on the challenge results.
- The authors use URLs as citations in the Introduction (e.g., http://www.cancerresearchuk.org/ and http://www.seer.cancer.gov/statfacts/html/prost.html and https://www.synapse.org/ProstateCancerChallenge). It seems that some of these should instead be references to peer-reviewed publications.
- The authors state that, "suggesting strong performance of the Halabi et al. model for identifying low- and high-risk mCRPC patients." What does this mean, more specifically, from a clinical standpoint?
- The author contributions section states, "assisted in the development of prediction models for treatment discontinuation." This doesn't seem relevant to this paper.
- I tried to install the R package dependencies that are described in the README file. However, it gave me an error message saying that some of the packages could not be found. To solve this, I had to specify a repository in the code ("repos" parameter of install.packages). It would be helpful if the authors changed this part of the code so that it will run out of the box.

I have read this submission. I believe that I have an appropriate level of expertise to confirm that it is of an acceptable scientific standard, however I have significant reservations, as outlined above.
